# Editorial: Novel SERS-Active Materials and Substrates: Sensing and (Bio)applications

**DOI:** 10.3389/fchem.2021.784735

**Published:** 2021-11-01

**Authors:** Piotr Piotrowski, Marcin Witkowski, Christa Brosseau, Yukihiro Ozaki, Agata Królikowska

**Affiliations:** ^1^ Faculty of Chemistry, University of Warsaw, Warsaw, Poland; ^2^ Department of Chemistry, Saint Mary’s University, Halifax, NS, Canada; ^3^ School of Biological and Environmental Sciences, Kwansei Gakuin University, Hyogo, Japan

**Keywords:** SERS (surface-enhanced Raman scattering), plasmonics and nanophotonics, nanosensors and nanobiosensors, hybrid nanocomposite, SERS applications, point of care (POC), Raman spectroscopy, rational design

Nearly 50 years have passed since the encounter of the surface-enhanced Raman scattering (SERS) phenomenon, which had a bumpy ride from a misinterpreted discovery to well-planned applications. SERS enhancement factors—defined as the intensity ratio between SERS and conventional Raman scattering signal for a given analyte normalized by the number of molecules probed—can typically achieve 8–10 orders of magnitude for the plasmonic substrates with inter-/intra-particle nanogap, while these values can exceed 10^11^, in case of, to name one example, cascaded nanooptical structures combining refractive and plasmonic optics (Kamp et al., 2020). However, reliable estimation of SERS enhancement factor, as well as fabrication of SERS-active materials and substrates guaranteeing reproducibility of SERS signal, controlled optical properties and interactions with the examined molecules, and viable quantitative analysis employing SERS spectroscopy are still the most challenging issues to overcome the limitations of SERS in order to become a routine analytical technique.

Recent years have been extremely advantageous to SERS spectroscopy, which, thanks to the development of nanotechnology and progress towards a higher level of the theory—in tandem with an improved detection sensitivity of Raman instruments and advances in computing power capacities—has grown to a role that goes beyond purely academic applications.

All of these, together with an enormous enhancement of the intrinsically weak Raman scattering signal, supported by high selectivity and specificity of the SERS method, offer simple detection and identification of the analyte of interest and use for designed applications.

Nowadays, a smart combination of computational approaches and vibrational spectroscopy aids the interpretation of SERS experimental results (Królikowska et al., 2020). On the other hand, a thoughtful design of innovative plasmonic nano-architectures, like those exploiting SERS-active three-dimensional volumetric materials (Szlachetko et al., 2020) or with engineered nanoparticle morphology, tailoring its SERS performance (Puente et al., 2021), boosts their real-life applicability. Properly customized plasmonic nanostructures can be successfully applied as SERS-active pH-sensitive nano- and microprobes (Piotrowski et al., 2014) or selective nanosensors of metabolites in body fluids (Zhang et al., 2020), act as multifunctional materials (Liu et al., 2021), as well as provide strategies for the goals as challenging as SERS-based chiral discrimination and identification (Wang et al., 2020) or point-of-care diagnostics (Clarke et al., 2017).

All these novel materials and analytical approaches allow SERS technique to reach beyond the laboratory and develop devices operating in real-life conditions, supporting industry and the development of (nano)technology, as well as being useful in biology, medicine and diagnostics.

This Research Topic contains seven articles: four research papers, two mini reviews, and one review, which cover a broad range of issues revolving around SERS-based sensing and its application in real-life problems.

Two of the original research papers included here focus on the development of new, high-performance substrates for SERS. Both studies represent the strong ongoing trend of steering away from single-component metallic plasmonic materials. Kasztelan et al. explore a novel substrate consisting of graphene oxide and silver nanoparticles (Ag NPs). By meticulous screening of the synthesis conditions, control on the composite parameters, such as the shape of Ag NPs and the material load with Ag NPs, can be achieved. Combination of the here-utilized SERS-supporting components leads to increased intensity of the signal and sheds some light on the mechanism and general properties of such hybrid materials.

On the other hand, Żygieło et al. approach the well-known issue of uncontrollable aggregation of hybrid magnetic-plasmonic nanoparticles, using Fe_3_O_4_/AgNPs as a proof of concept. There, improved stability is achieved by encapsulation of the material in nanosilica, whose generation is facilitated by 2-mercaptoethanesulfonate ions acting as both a SiO_2_ primer and a Raman tag. Improved stability and longevity of such nanotags increase the feasibility of real-world applications (security tags on banknotes/maintaining activity in aggressive bio-environment, e.g., yeast suspension). Thorough optimization of the fabrication process and comprehensive characterization of the final product will help other researchers translate the results into their own studies.

The work of Dusa et al. constitutes a valuable input into substrate engineering from the theoretical side using finite-difference time-domain (FDTD) simulations. Various combinations of nanoparticles’ systems are considered (dimers of Au NPs, dimers of Au nanorods and the respective monomers) in the context of SEHRS (surface-enhanced hyper Raman scattering), in a broad range of excitation (from 540 to 1,800 nm). Determined enhancement conditions are essential to properly select the right system for use in this method—complementary to SERS spectroscopy and valuable in the studies of biological systems.

The final original research by Tay et al. covers the study on a novel paper-based sensor of narcotic drugs. Right functionalization strategy, in this case with iodide ions, improves the sensor’s performance by obtaining stronger signals and quicker detection. The ease of fabrication, using inkjet printing, is a strong advantage in the commercialization process of such devices. Moreover, such prepared nanosensors can be straightforwardly used in field applications.

Significance of developing efficient and reliable SERS-based sensors is emphasized in the mini-reviews from this Research Topic. Szaniawska and Kudelski survey the current studies on the determination of biochemically and medically relevant species. They highlight, among others, glucose detection and intracellular SERS as hot topics and point out towards combination with other techniques, like polymerase chain reaction (PCR) or paper-based sensors, the latter being perfectly illustrated by the research of Tay et al. mentioned above.

Urgency of application of SERS spectroscopy in medical diagnosis is carefully explained in the mini review by Quarin and Strobbia. They present recent advances towards the SERS-based point-of-care diagnosis. Such a form of disease recognition promotes fast treatment and prevents disease spread, which is especially important in today’s world. They take into consideration various SERS-sensing mechanisms and their advantages in the point-of-care approach. The fact that most of the reviewed methods are based on the detection of various species of microRNA, which are markers for numerous diseases, is deemed extremely helpful.

Finally, Xi and Liang provide an overview of the ongoing efforts to bring SERS closer to the patients, as they review clinical trials which employ Raman or SERS spectroscopy. It turns out that such studies have been recorded in Official Registries in many countries around the globe, including Japan, Germany, China, India and Australia. Various methods listed there (FT-Raman, confocal Raman, resonance Raman etc.) indicate the rising appreciation outside of the community for the increasing availability of Raman-based techniques. A long list of diseases, including diabetes, pulmonary diseases and various cancer types, is a promising sign in the context of future applications.

We are sure that the reader of this Research Topic will be provided with the most current insight into the development of SERS sensing investigations and the mixed bunch of papers herein will introduce them to its current application studies, as well as inspire them to implement their own ideas in the field.
